# Stroke Damage Is Exacerbated by Nano-Size Particulate Matter in a Mouse Model

**DOI:** 10.1371/journal.pone.0153376

**Published:** 2016-04-12

**Authors:** Qinghai Liu, Robin Babadjouni, Ryan Radwanski, Hank Cheng, Arati Patel, Drew M. Hodis, Shuhan He, Peter Baumbacher, Jonathan J. Russin, Todd E. Morgan, Constantinos Sioutas, Caleb E. Finch, William J. Mack

**Affiliations:** 1 Zilkha Neurogenetic Institute, Keck School of Medicine, University of Southern California, Los Angeles, CA, United States of America; 2 Department of Neurosurgery, Keck School of Medicine, University of Southern California, Los Angeles, CA, United States of America; 3 Davis School of Gerontology, University of Southern California, Los Angeles, CA, United States of America; 4 Viterbi School of Engineering, University of Southern California, Los Angeles, CA, United States of America; 5 Department of Neurobiology, University of Southern California, Los Angeles, CA, United States of America; Indiana School of Medicine, UNITED STATES

## Abstract

This study examines the effects of nano-size particulate matter (nPM) exposure in the setting of murine reperfused stroke. Particulate matter is a potent source of inflammation and oxidative stress. These processes are known to influence stroke progression through recruitment of marginally viable penumbral tissue into the ischemic core. nPM was collected in an urban area in central Los Angeles, impacted primarily by traffic emissions. Re-aerosolized nPM or filtered air was then administered to mice through whole body exposure chambers for forty-five cumulative hours. Exposed mice then underwent middle cerebral artery occlusion/ reperfusion. Following cerebral ischemia/ reperfusion, mice exposed to nPM exhibited significantly larger infarct volumes and less favorable neurological deficit scores when compared to mice exposed to filtered air. Mice exposed to nPM also demonstrated increases in markers of inflammation and oxidative stress in the region of the ischemic core. The findings suggest a detrimental effect of urban airborne particulate matter exposure in the setting of acute ischemic stroke.

## Introduction

Clinical and population based studies have established an association between acute stroke mortality and air pollution [1, 2]. A recent investigation suggests that exposure to levels of particulate matter considered safe by the US Environmental Protection Agency increases the risk of ischemic stroke within hours of exposure [3]. Elevated post-stroke mortality has been documented in individuals living in close proximity to high-traffic roadways [4]. These studies establish a relationship between air pollution exposure and acute stroke, consistent with associations previously noted in cardiovascular disease [5–7]. Systematic reviews have demonstrated associations between PM_2.5_ and PM_10_ exposures and higher total cerebrovascular disease mortalities and established a concentration-response relationship between both short and long-term PM_2.5_ exposure and stroke [2], [8]. Further, studies have suggested a temporal association between gaseous/ particulate air pollutants and admissions to hospital for stroke and mortality from stroke [9]. To date, however, no experimental studies have examined the relationship between exposure to air pollution and severity of damage resulting from stroke. Air pollution is a prevalent environmental source of both inflammation and oxidative stress, processes contributory to the progression of stroke [10, 11]. Nanoparticulate matter (nPM) derived from vehicular exhaust may exacerbate cerebral ischemia/ reperfusion injury via upregulation of inflammatory mediators and generation of oxygen free radicals, resulting in regional microvascular failure. A recent experimental study established an association between season-dependent particulate matter levels and ischemia-like neuronal injury in-vitro. Further, the investigation demonstrated endothelial dysfunction, inflammation, and functional impairment secondary to particulate matter exposure (strongest in the winter month sample) in an experimental rat model [12]. This investigation leverages an experimental murine model of cerebral ischemia/ reperfusion to examine the impact of a pervasive environmental exposure on the progression and severity of brain injury following acute ischemic stroke. The study is the first to examine the effect of nPM on the progression and evolution of acute ischemic stroke.

## Materials and Methods

### Protocol

All procedures utilized in this study were approved by the Institutional Animal Care and Use Committee (IACUC; protocol # 11968) of the University of Southern California and carried out in accordance with the Guide for the Care and Use of Laboratory Animals (NIH). All mice were male C57BL/6J mice (15–16 weeks of age; 24-29g) and housed in a barrier facility with free access to food and water on a 12-hour light dark cycle, except during the nPM/ filtered air exposures. The mice did not have access to food and water during the five-hour exposure periods.

#### Particulate matter collection

Nano-size particulate matter was collected in an urban area in central Los Angeles, impacted mostly by traffic emissions, and administered as previously described [13, 14]. Briefly, urban nPM (aerodynamic diameter <200 nm) was obtained at 400 L/min flow using a high-volume ultrafine particle sampler [14]. The sampler incorporates an ultrafine particle multiple rectangular (slit) geometry jet conventional impactor and an after-filter on which nanoparticles are collected [14]. nPM was collected at an urban site situated adjacent to the CA-110 highway in Los Angeles [13]. Aerosols represent a mix of fresh ambient PM mostly from vehicular traffic nearby this freeway [15]. The impactor and after-filter holder system employs high flow rates under very low pressure drops, allowing for animal exposure to ultrafine aerosols at near atmospheric pressure and at significantly higher flow than the typical human breathing rates [14]. The nPM was collected on pretreated Teflon filters (8x10”, PTFE, 2 μm pore; Pall Life Sciences, Port Washington, NY) and then transferred into aqueous suspension by 30 min soaking of filters in Milli-Q deionized water (resistivity, 18.2 MW; total organic compounds < 10 ppb; particle free; bacteria levels < 1 endotoxin units/mL; endotoxin-free glass vials), followed by vortexing (five min), sonication (30 min) and resuspension. No endotoxin has been detected in these suspensions (*Limulus* amebocyte lysate assay: LPS <0.02EU/ml). As a control, fresh sterile filters were sham extracted and stored. Aqueous nPM suspensions were pooled and frozen as a stock at –20°C, which retains chemical stability for greater than or equal to three months [16, 17].

#### Particulate matter exposure

Mice were transferred to whole-body exposure chambers as described previously by our group [13, 18–20]. Temperature and airflow were controlled for adequate ventilation and to minimize buildup of animal-generated contaminants. Mice were randomized and either re-aerosolized nPM or filtered air was delivered to the sealed exposure chambers for five hours/day, three-days/ week for three weeks.

A VORTRAN atomizer was used to re-aerosolize the nano-particulate suspensions from the high-volume sampler discussed earlier, using compressed particle-free filtered air. The approach was identical to that described in Morgan et al (13). Passage through a silica gel diffusion-dried the generated nanoparticles and static charges were removed by passage over polonium-210 neutralizers. Particle size and concentration (target mass concentrations were in the range of 300–350 μg/m^3^- roughly twice as high levels as a busy freeway) [21] were continuously monitored by a scanning mobility particle sizer (SMPS model 3080; TSI Inc., Shoreview, MN) in parallel with the animal exposure chambers. From the total of 15 l/min of aerosol flow generated, the majority (10 l/min) was drawn through the exposure chamber. The remaining 5 l/min was diverted to filters for particle collection and characterization. The mass concentration of the nPM was determined by pre- and post- weighing under controlled temperature and relative humidity. Teflon and quartz filters, sampled concurrently the exposure aerosol during the experiments [16, 17]. The composition of nPM was monitored. Inorganic ions [ammonium (NH_4_^+^), nitrate (NO_3_^–^), sulfate (SO_4_^2–^)] were analyzed by ion chromatography and PM-bound metals/ trace elements assayed by magnetic-sector inductively coupled plasma mass spectroscopy. Elemental and organic carbon (EC, OC) were also quantified by the NIOSH (National Institute for Occupational Safety and Health) thermal optical transmission method performed on the quartz filters, as described in by Schauer et al. (2003) [22]. The analytical approaches for measuring the inorganic and organic compound contents of these samples have been previously described [16, 17].

The particulate matter was obtained from two separate collections. Each was administered equally to the two treatment cohorts. A detailed physical and chemical characterization of the collected and re-aerosolized nanoparticles from each collection cycle was performed as discussed in an earlier publication [13].

#### Murine middle cerebral artery occlusion model

One day after nPM exposures, mice underwent middle cerebral artery occlusion/ reperfusion. Studies used the intraluminal filament model described previously, with minor modifications [23]. Briefly, mice were anesthetized with four percent isofluorine at induction and maintained at two percent isofluorine during the procedure. Rectal temperature was maintained at 35.5°C. Middle cerebral artery occlusion (MCAO) was performed by advancing a silicone-coated, monofilament (Doccol, Sharon, MA; 6–0 0.23mm diameter) to the right middle cerebral artery origin. Following 35 minutes of ischemia, the occluding filament was withdrawn to allow for reperfusion. Transcranial cerebral blood flow was measured using Laser Doppler Flowmetry (Periflux system PF 5010, Perimed, Inc, Jarfalla, Sweden) with 0.5mm flexible fiberoptic Doppler probes (Perimed, Jarfalla, Sweden) attached to intact skull 1mm posterior and 5mm lateral (right) to the bregma. Strict criteria were used to prospectively exclude animal that did not experience adequate CBF dropoff (>55% baseline). Upon recovery, mice were given 100μl of Carprofen (1mg/ml) S.C, and housed for 24 hours.

#### Measurement of cerebral blood flow

Two minute LDF recording tracings were taken for each animal in the prone and supine positions to establish baseline CBF. Two-minute LDF recordings were taken after CCA ligation after ECA ligation and after ICA clipping. LDF recording was maintained throughout the 35-minute duration of MCAO. Re-perfusion recordings were taken for two minutes following the removal of the suture and return to the prone position.

#### Neurologic deficit scores

Neurologic Deficit scores (0–28 scale) were obtained 24 hours after the procedure. Scoring was performed by two blinded observers and averaged for a final score on each mouse.[24]

#### Infarct volume

Infarct volume was assessed at 24 hours post-ischemia. Following neurological examination, mice were euthanized, and 2-mm sections of brain were stained with TTC. Digital images were captured and Image J software (NIH, Bethessda, MD) was used to determine quantitative infarct volume by an observer blinded to the identity of individual animals. Corrected infarct volume was calculated according to the following equation by two independent, blinded observers:

$$Corrected\;infarct\;volume\;(CIV)\%=\frac{\left[Contralateral\;hemisphere\;volume-(Ipsilateral\;hemisphere\;volume)\right]}{Contralateral\;hemisphere\;volume}\times 100$$

#### Immunohistochemistry

Primary antibodies and reagents used for immunohistochemistry were commercially available. Mice underwent focal cerebral ischemia and were euthanized after 24 hours. Following transcardiac PBS perfusion, brains were rapidly harvested, fixed in 4% paraformaldehyde for twenty-four hours, and cryoprotected. Brains were frozen in Optimal Cutting Temperature Compound, placed at -80°C and cut using a cryostat (20 micron coronal sections). Antigen retrievel was performed by citrate buffer (pH 6.0) for a total of ten minutes in the microwave (five cycles of 2 minutes). Samples were then washed and sections were blocked with the secondary antibody–appropriate serum (ten percent donkey serum) with 0.2% Triton X-100 for 60 minutes at room temperature. Primary antibodies were diluted in PBS containing 0.2% Triton X-100. Sections were treated with primary antibodies anti-Ly-6G (rat 1:500; Ebioscience, San Diego, CA; catalog number 14–5931), anti-8OHG (goat 1:500; Abcam, Cambridge, UK; ab108020), anti-C5 (mouse 1:50 Hycult Biotech, Netherlands; clone BB5.1), anti-C5a (goat 1:50 Santa Cruz Biotechnology, Dallas, TX; SC-21941), anti CD88 (rat 1:200 Biolegend, San Diego, CA; 135802), anti-p47^phox^ (goat 1:200 Santa Cruz Biotechnology, Dallas, TX; SC-7660), anti- gp91^phox^ (goat 1:200 Santa Cruz Biotechnology, Dallas, TX; SC-5827), and anti-MAP2 (rabbit 1:500; Sigma-Aldrich, St. Louis, MO; catalog number M3696) for immunofluorescent staining overnight. Sections were washed and incubated with secondary antibodies (Alexa fluorochrome 488 or 568, Invitrogen, Carlsbad, CA) for one hour, nuclei were stained with DAPI (Invitrogen), mounted and coverslipped. Slides were then visualized using Zeiss 510 confocal microscopy and BZ-9000 fluorescent microscopy (Keyence, NJ). For all immunofluorescence studies, controls were performed without the use of the primary antibodies (negative controls).

Necrotic core was identified by regional loss of MAP-2 immunopositivity, which has been documented as an early immunochemical marker of ischemic neuronal injury [25]. Cells were identified as neurons based on morphology/size on light microscopy and presence of remnant MAP-2 immunopositivity

Semiquantitative analysis of immunohistochemistry was performed to assess total Ly-6G, 8-hydroxyguanosine, C5, CD88, p47^phox^, and gp91^phox^immunopositivity using NIH Image J software. Multiple (5) nonoverlapping high-power fields were imaged. Adobe Photoshop version 5.5 was used to acquire and process the images, which were then analyzed using Image Pro-Plus 4.5 (Media Cybernetics, Silver Spring, MD) software. Cell count (Ly-6G) or density area occupied by positive immunostaining (8-hydroxyguanosine, C5, C5a, CD88, p47^phox^, gp91^phox^) was calculated for each image, and mean value for each animal was determined by averaging values from all images taken from that animal.

#### Assessment of infiltrating granulocytes

Mice exposed to nPM or filtered air underwent focal cerebral ischemia/ reperfusion surgery as described above and were euthanized after 24 hours. Tissue sections were prepared as detailed above using anti–Ly-6G primary antibody. Ly-6G was quantified to assess granulocyte infiltration into the stroke region. Semiquantitative analysis of immunohistochemistry was performed to assess total Ly-6G immunopositivity using NIH Image J software. An observer blinded to the identity of the mice counted Ly-6G cells in five representative medium-power fields (40X objective). The fields were chosen in the ischemic region and contralateral hemisphere in an anatomically consistent manner between mice. Results are reported as mean number of positive cells per field. The mean value for each animal was determined by averaging values from all images taken from that animal.

#### Assessment of oxidative stress

Mice exposed to nPM or filtered air underwent focal cerebral ischemia/ reperfusion surgery as described above and were euthanized after 24 hours. Tissue sections were prepared as detailed above using anti–8-hydroxyguanosine primary antibody. Semiquantitative analysis of immunohistochemistry was performed to assess total area occupied by positive 8-hydroxyguanosine staining using NIH Image J software. An observer blinded to the identity of the mice measured staining density in five representative medium-power fields (40X objective). The fields were chosen in the ischemic region and contralateral hemisphere in an anatomically consistent manner between mice. Results are reported as intensity of staining density in both ipsilateral and contralateral hemispheres. The mean value for each animal was determined by averaging values from all images taken from that animal.

#### Assessment of inflammation/ reactive oxygen species

Mice exposed to nPM or filtered air underwent focal cerebral ischemia/ reperfusion surgery as described above and were euthanized after 24 hours. Tissue sections were prepared as detailed above using anti–C5, C5a, CD88, p47^phox^ and gp91^phox^ primary antibodies. Semiquantitative analysis of immunohistochemistry was performed to assess total area occupied by positive C5, C5a, CD88, p47^phox^, and gp91^phox^ staining using NIH Image J software. An observer blinded to the identity of the mice measured staining density in five representative medium-power fields (40X objective). The fields were chosen in the ischemic region and contralateral hemisphere in an anatomically consistent manner between mice. Results are reported as intensity of staining density in both ipsilateral and contralateral hemispheres. The mean value for each animal was determined by averaging values from all images taken from that animal.

### Statistical analyses

Between-group differences were compared using two-tailed unpaired Student’s t-tests for continuous variables and non-parametric, Mann-Whitney tests for ordinal scales. Data were presented as mean±SD (normally distributed) or median and interquartile range (non-parametric). P ≤0.05 was considered statistically significant.

## Results

### Particulate matter composition

**Fig 1:**
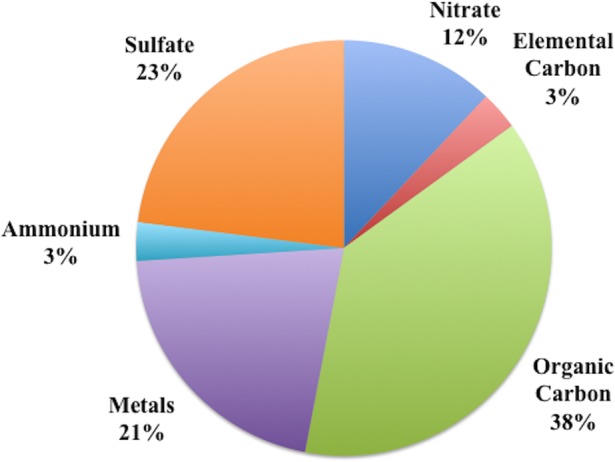
Percent fraction of nPM chemical components (including organic carbon, elemental carbon, ions and metals) during the exposure period.

A detailed physical and chemical characterization of the collected and re-aerosolized nanoparticles is depicted in Fig 1 and Table 1. The average over the exposure period mass and number concentrations were 343 ±30.6 (μg/m^3^) and 5.6 x 10^4^ ±1.1 10^4^ particles /cm^3^, respectively. The geometric mean particle diameter was 59 ±7.2 nm. Regarding chemical composition, as shown in Fig 1, organic carbon was the most predominant species, accounting for about 38% of the total mass, with sulfate being the second most predominant element. Trace elements and metals contributed about 21% to the total nPM mass. The concentrations of the most important metals and elements are shown in Table 1. Table 2 illustrates the chemical characterizations of the two collection periods. The compositions of the nPM collected during these periods were very consistent.

**Table 1:** Concentration of selected metals (ng/m^3^) during the exposure.

Metal	Concentration (ng/m^3^)*
Na	12132±360
Mg	1701±89
Al	168±10.3
K	3214±346
Ca	9806±521
Ti	0.62±0.59
V	36.36±1.81
Cr	5.76±0.59
Mn	98.44±4.8
Fe	97.07±6.52
Ni	28.13±2.06
Cu	381.4±17.8
Zn	919.2±48.0
Mo	15.26±0.47
Cd	2.91±0.26
Ba	274.4±17.2
Pb	8.92±0.34

*Errors are expressed as standard deviations

**Table 2:** Collection times and concentrations of the two collection periods.

Parameter	Period I	Period II
**Particle collection period**	Dec 2013-Mar 2014	Mar 2014-Apr 2014
**Mass concentration (μg/m**^**3**^)^*^	343 (±30.6)	304 (±42.2)
**Number concentration (#/cm**^**3**^**)**	5.6 E^4^ (±1.1 E^4^)	4.1 E^4^ (±0.93 E^4^)
Mode Diameter (nm)^*^	59 (±7.2)	(±4.7)

*Errors are expressed as standard deviations

### Murine stroke

27 mice (14 nPM, 13 filtered air) were randomized for treatment. Two mice (1nPM, 1 filtered air died of anesthetic/ procedural complications prior to ischemia. 25mice (13 nPM, 12 filtered air) underwent middle cerebral artery ischemia/ reperfusion. The perioperative mortality of this cohort was 8% (1/13) in the nPM cohort and 0% (0/12) in the filtered air cohort. All underwent LDF measurements and neurological outcome assessments. Five mice were excluded from the LDF reperfusion measurement results due to technical acquisition difficulties at one or more time points. Each of these mice did demonstrate adequate CBF dropoff during suture insertion. The nPM mouse died prior to behavioral testing, sacrifice, and infarct volume measurement (delayed staining demonstrated a large, hemispheric stroke). This mouse was not included in infarct volume or behavioral analysis. Following sacrifice, brains from 17 (8 nPM, 9 filtered air) were harvested for stroke volume measurements and six (3 nPM, 3 filtered air) for immunohistochemistry (assessments of inflammation and oxidative stress).

There were no significant differences in animal weight between the nPM and filtered air cohorts. Continuous transcranial Doppler recordings of CBF demonstrated equivalent blood flow between groups at all baseline and occlusion time points (Table 3). There were no statistical differences in the number of animals in each group that were excluded for failure to meet CBF criteria (n = 0).

**Table 3:** Demographics and Experimental Data.

**Demographics**		
	**nPM**	**Filtered**
Strain	C57 Black 6J	C57 Black 6J
Age	15–16 Weeks	15–16 Weeks
Mean Initial Weight	27.72 ± 1.21	27.38 ± 1.66
**Experimental Data**		
	**nPM**	**Filtered**
Sample Size (n)	13	12
Postoperative Mortality	1 (8%)	0 (0%)
% CBF Decline	-78.54 ± 9.95%	-78.25 ± 11.36%

### Neurological outcomes

**Fig 2:**
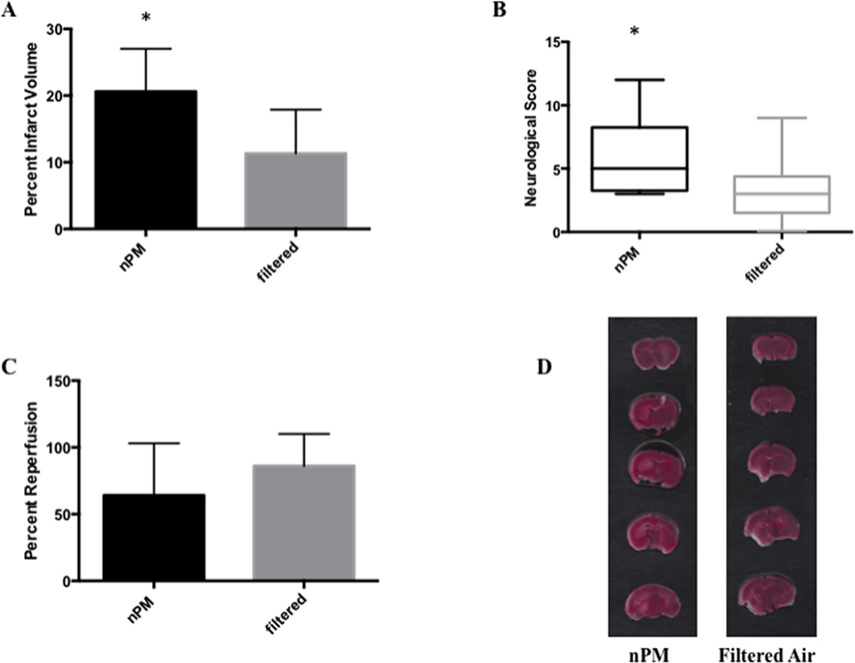
Infarct volume, neurological function and reperfusion following murine stroke. Animals were assessed at 24 hours post ischemia. Cerebral infarct volumes are larger (A), and neurological deficit scores increased (B) in mice exposed to nPM when compared to those exposed to filtered air. Percent reperfusion differences do not differ significantly between the two groups (C). * signifies p<0.05. Representative infarcts are shown in panel (D)

Following cerebral ischemia/ reperfusion, mice exposed to nPM demonstrated larger infarct volumes [20.6 ±6.4% (n = 8) vs. 11.3 ±6.6% (n = 9); p = 0.018] and less favorable neurological deficit scores [5.0, IQR 3.25–8.25, (n = 12) vs. 3.0, IQR 1.50–4.375, (n = 12); p = 0.015] when compared to mice exposed to filtered air (Fig 2A and 2B).

### Reperfusion

LDF analysis did not demonstrate significant differences reperfusion in the cohort of mice exposed to nPM (64 ±39%, n = 10) when compared to those exposed to filtered air (86 ±24%, n = 10, p = ns; Fig 2C)

### Ly-6G positive cell count

**Fig 3:**
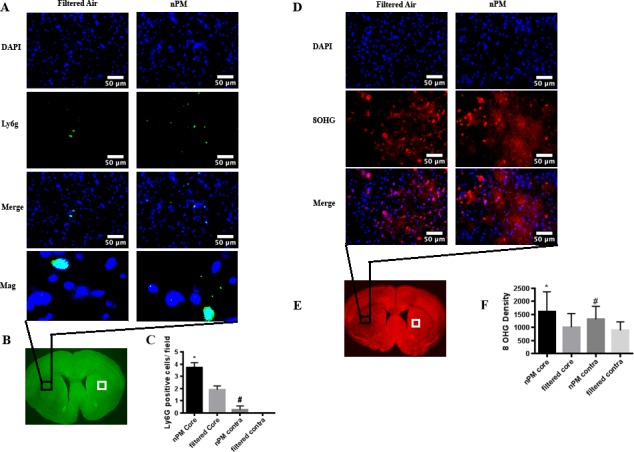
Semiquantitative immunohistochemical analysis demonstrates infiltrating granulocytes and oxidative stress are increased in mice exposed to nPM. Left: Filtered air and nPM exposed mice euthanized at 24 hours and stained for Ly-6G (green) in the ischemic region. Nuclei (DAPl) are stained in blue (A). Low magnification representation of region analyzed (B). Graphical representation of granulocyte cell count per high-powered field (40X objective) for filtered air and nPM exposed mice in the ischemic region and contralateral hemispheres (C). Right: Filtered air and nPM exposed mice euthanized at 24 hours and stained for 8-hydroxyguanosine (red) in the ischemic region. Nuclei (DAPI) are stained in blue (D). Low magnification representation of region analyzed (E). Graphical representation of 8-hydroxyguanosine densities per high-powered field (40X objective) for filtered air and nPM exposed mice in the ischemic region and contralateral hemisphere (F). *Signifies comparison of ipsilateral hemisphere counts (P<0.05). #Signifies comparison of contralateral hemisphere counts (P<0.05). Scale bar: 50 μm.

Following cerebral ischemia/ reperfusion, mice exposed to nPM demonstrated significantly higher Ly-6G positive cell counts in the ischemic core [3.7± 0.42(n = 3);] when compared to mice exposed to filtered air (1.9±0.31 (n = 3); p<0.05). Further, the mice exposed to nPM demonstrated significantly higher Ly-6G counts [0.263±0.31 (n = 3)] in the contralateral hemisphere than did the mice exposed to filtered air [0.05±0.0 (n = 3); p<0.05; Fig 3].

### 8-hydroxyguanosine density

Following cerebral ischemia/ reperfusion, mice exposed to nPM demonstrated significantly higher 8-hydroxyguanosine densities in the ischemic core [1614.68±747.49 (n = 3)] when compared to mice exposed to filtered air [1013.88±517.85 (n = 3); p<0.05]. Further, the mice exposed to nPM demonstrated significantly higher 8-hydroxyguanosine densities in the contralateral hemisphere [1317.08±493.73 (n = 3)] than did the mice exposed to filtered air [888.51±323.13 (n = 3); p<0.05; Fig 3].

### Complement C5, C5a, C5a receptor density

**Fig 4:**
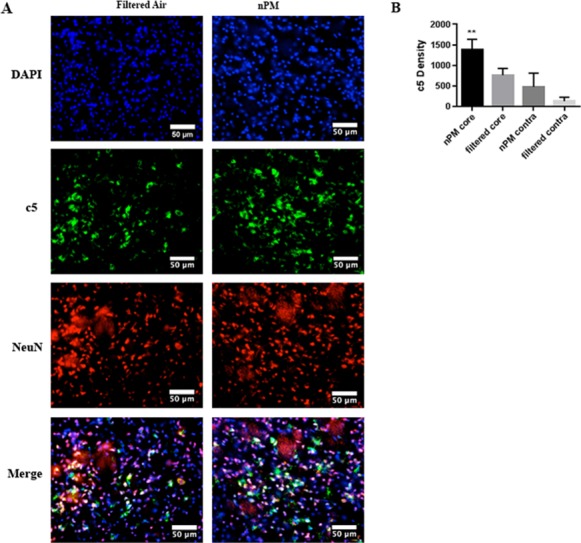
Semiquantitative immunohistochemical analysis demonstrates C5 is increased in mice exposed to nPM. Filtered air and nPM exposed mice euthanized at 24 hours and stained for C5 (green) in the ischemic region. Nuclei (DAPI) are stained in blue. Neurons are stained in red (A). Graphical representation of C5 density per high-powered field (40X objective) for filtered air and nPM exposed mice in the ischemic region and contralateral hemispheres (B). **Signifies comparison of ipsilateral hemisphere counts (P<0.01). Scale bar: 50 μm.

Following cerebral ischemia/ reperfusion, mice exposed to nPM demonstrated significantly higher complement C5 densities in the ischemic core [1378.18 ±127.97 (n = 3)] when compared to mice exposed to filtered air [761.29 ±82.93 (n = 3); p<0.01; Fig 4]. C5 deposition was most evident on neurons (Fig 4A).

**Fig 5:**
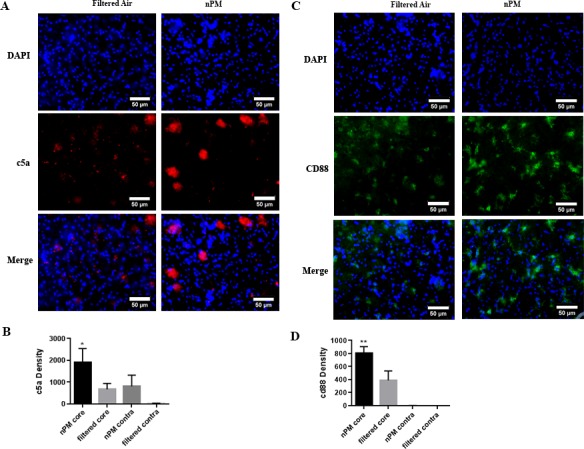
Semiquantitative immunohistochemical analysis demonstrates C5a and C5a receptor (CD88) are increased in mice exposed to nPM. Left: Filtered air and nPM exposed mice euthanized at 24 hours and stained for C5a (red) in the ischemic region. Nuclei (DAPI) are stained in blue (A). Graphical representation of C5a density per high-powered field (40X objective) for filtered air and nPM exposed mice in the ischemic region and contralateral hemispheres (B). Right: Filtered air and nPM exposed mice euthanized at 24 hours and stained for C5a receptor (CD88) (green) in the ischemic region. Nuclei (DAPI) are stained in blue (C). Graphical representation of CD88 densities per high-powered field (40X objective) for filtered air and nPM exposed mice in the ischemic region and contralateral hemisphere (D). *Signifies comparison of ipsilateral hemisphere counts (P<0.05). **Signifies comparison of ipsilateral hemisphere counts (P<0.01). Scale bar: 50 μm.

Mice exposed to nPM demonstrated significantly higher complement C5a [1905.00 ±365.40 (n = 3)] and C5a receptor (CD88) [805.06 ±49.34 (n = 3)] densities in the ischemic core when compared to mice exposed to filtered air [669.19 ±157.23 (n = 3); p<0.05; 387.21 ±72.31 (n = 3); p<0.01, respectively; Fig 5].

### gp91^phox^ / p47^phox^density

**Fig 6:**
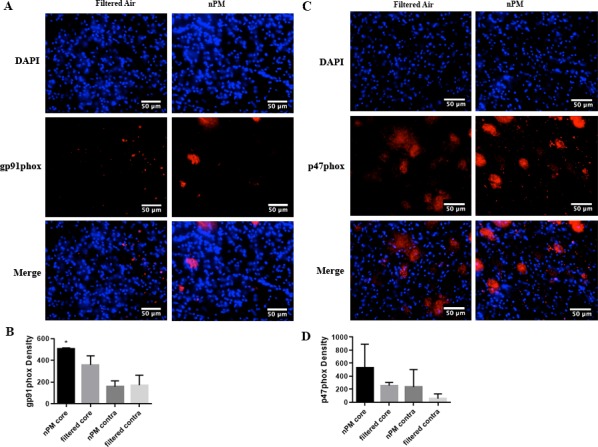
Semiquantitative immunohistochemical analysis demonstrates gp91^phox^ is increased in mice exposed to nPM. Left: Filtered air and nPM exposed mice euthanized at 24 hours and stained for gp91^phox^ (red) in the ischemic region. Nuclei (DAPI) are stained in blue (A). Graphical representation of gp91 density per high-powered field (40X objective) for filtered air and nPM exposed mice in the ischemic region and contralateral hemispheres (B). Right: Filtered air and nPM exposed mice euthanized at 24 hours and stained for p47^phox^ (red) in the ischemic region. Nuclei (DAPI) are stained in blue (C). Graphical representation of p47^phox^ densities per high-powered field (40X objective) for filtered air and nPM exposed mice in the ischemic region and contralateral hemisphere (D). *Signifies comparison of ipsilateral hemisphere counts (P<0.05). Scale bar: 50 μm.

Following cerebral ischemia/ reperfusion, mice exposed to nPM demonstrated significantly higher gp91^phox^ densities in the ischemic core [502.51 ±7.31 (n = 3)] when compared to mice exposed to filtered air [357.27 ±48.45 (n = 3); p<0.05]. p47^phox^ densities did not differ significantly between the two cohorts (Fig 6).

## Discussion

Recent systematic reviews and meta-analyses have demonstrated associations between particulate matter exposure and stroke incidence/ mortality [2, 8, 9]. To date, however, no studies have directly examined the effect of nPM on stroke progression and severity. Our data suggests that subchronic nPM exposure results in larger infarct volumes and less favorable neurological outcomes following reperfused stroke. This is the first study to characterize the effects of nPM in an experimental stroke model. The findings suggest a detrimental effect of urban airborne particulate matter exposure in the presence of large vessel acute ischemic stroke. Importantly, these findings implicate nPM exposure as a modifiable risk factor impacting the size and severity of acute stroke.

Morgan et al. have previously demonstrated that exposure to nanoscale particulate matter derived from urban traffic results in glutamanergic-mediated neurotoxicity in rodent microglia and neurons both in-vitro and in-vivo [13]. These findings are consistent with other recent studies, suggesting that exposure to particulate matter generates inflammation and oxidative stress in multiple brain regions [13, 26–31]. Rodent nPM exposure studies have documented increases in inflammatory cytokines/ chemokines and immune-related transcription factors [29, 31]. These are the very processes known to be detrimental to the rescue of hypoperfused tissue in the setting of acute stroke. Upregulation of regional inflammatory mediators can enhance susceptibility to the neurotoxic effects of stroke by increasing the density of reactive astrocytes and microglia. When primed, these cells may promote further inflammation. In-vitro studies demonstrate that diesel exhaust particles promote release of oxygen free radicals from brain microglia, resulting in superoxide-mediated cellular injury [32]. Similarly, post-mortem brain tissues from canines exposed to high levels of air pollution reveal tissue injury consistent with oxidative processes [33]. Our experiments demonstrate increases in infiltrating granulocyte number and 8-hydroxyguanosine densities in the ischemic core, implicating both inflammatory and oxidative processes. As these concentrations are measured in the core infarct region, they are likely not dependent on the overall size of the stroke. Not surprisingly, both Ly-6G and 8-hydroxyguanosine levels were also significantly higher in the contralateral hemispheres of mice exposed to nPM when compared to those exposed to filtered air. These differences suggest elevated baseline levels of inflammation and oxidative stress secondary to particulate matter exposure. Such global changes may prime regional capillary beds for microvascular failure in the setting of acute stroke.

Our studies also examined inflammatory mediators (complement cascade) and markers of reactive oxygen species/ NADPH oxidases following ischemia/ reperfusion in mice exposed to nPM and filtered air. Mice exposed to nPM demonstrate increased levels of C5, C5a, and C5a receptor (CD88) when compared to those exposed to filtered air. Further, gp91^phox^ levels were significantly elevated in the ipsilateral hemisphere (stroke region) of mice exposed to nPM.

For comparison, we cite prior data from a separate study without experimental stroke which examined markers of oxidative stress. In the cerebral cortex after forty-five hours of nPM, TNFa mRNA and protein were elevated, whereas no changes were evident in 4-hydroxynonenal (lipid peroxidation) and 3-nitrotyrosine (nitrosative stress) [34]

The complement cascade is a phylogenetically ancient component of the immune system, composed of more than thirty regulatory proteins. Sequential cleavage generates C3a and C5a, potent anaphylotoxins which can contribute to microvascular failure through generation of downstream inflammatory mediators. Prior studies have demonstrated a role for complement-mediated injury in experiemntal stroke and chronic cerebral hypoperfusion [35, 36]. Experiments have focused on the role of the anaphylotoxins. Ischemic protection is evident in C3 knockout mice and through pharmacologic inhibition of the C3a and C5a receptors following experimental ischemia/ reperfusion injury[36–38]. Further, C5 deficiency is protective in the setting of murine chronic cerebral hypoperfusion[39]. In the current experiments, increased deposition of C5, C5a and C5 receptor (CD88) in mice exposed to nPM suggests a potential role for complement mediated inflammation and recruitment of penumbral tissue following acute ischemic stroke.

The effects of microvascular failure on stroke progression are well documented. Imaging studies demonstrate that marginally viable cortical tissue is recruited into a central ischemic core within forty-eight hours of cerebral large vessel occlusion. Moreover, susceptible territories closely match early perfusion deficits, implying that regional flow failure is a prominent feature of tissue ultimately destined for infarction. Such progression strongly correlates with unfavorable neurological outcomes in the clinical setting [40]. The regional cellular environment is critical during hours following acute ischemic stroke. The presence of nanoparticulate matter at the time of vessel occlusion can render the brains adaptive processes less effective. Cerebral reperfusion is beneficial. However, coupled with proinflammatory changes and oxidative stress, re-establishment of blood flow can engender detrimental alterations in the local microvascular milieu [41–43]. These reactions shift the intrinsic hematologic balance from fibrinolysis to coagulation, and lead to capillary plugging and thrombosis. Together these alterations result in microvascular failure, generating further recruitment of marginally viable neurons into the ischemic core.

There is no significant difference in reperfusion between the nPM and filtered air cohorts at the level of the laser Doppler flowmetry probes (cortical surface of right MCA territory). Regional blood flow differences and/ or measurement capacity may contribute to this finding. Further, there are a vast number of different mechanisms/ pathways by which nPM exposure might impact stroke progression. Among these are microvascular failure, apoptosis, reperfusion injury, microhemorrhages, and cortical spreading depolarization.

The study examines nPM exposure immediately prior to ischemic stroke onset. The twenty-four hour assessment point was selected to delineate acute stroke progression and penumbral recruitment into the ischemia core. This endpoint eliminates the cofounding influences that may impact subacute and chronic recovery or regeneration. However, this also restricts the capacity to examine delayed functional outcomes and does not assess the impact of exposure during stroke recovery. Future studies can incorporate additional time points to determine the temporal evolution of the infarcted tissue. Further, this investigation employs a subchronic exposure paradigm. We aimed to assess a relatively short exposure duration, likely to demonstrate clinical effect. As nPM has not been previously studied in experimental stroke models, exposure durations are based on data generated from pilot studies and other paradigms in our laboratory [13].

The exposure employed in this study is an aerosol that has substantial similarities in size and chemical composition to that typical in an urban area, at highly increased but still environmentally realistic, exposure levels. Exposure levels of about 300 μg/m^3^ are comparable to those in a busy Los Angeles freeway heavily impacted by heavy duty diesel trucks [44, 45].

While water-soluble species are captured at 100%, insoluble PM species are not captured as well due to loss of volatiles and semivolatile organic compounds. Clearly, some toxicity is missed as a result of the lower insoluble PM content. However, our overall effects seen in vivo and in vitro, at relatively realistic exposure scenarios, are clear. An advantage of this approach, which is not available when using concentrators, is a consistent aerosol across the entire exposure period. This is not a possibility when working with real world PM, which varies temporally and spatially.

These experiments do not attempt to distinguish the mechanism by which nPM exposure affects the brain (e.g.: directly via transport via the olfactory nerve, indirectly through the systemic inflammatory markers, or indirectly through infiltration of peripheral monocytes). Our experimental design aims to assess the impact of nPM exposure on stroke progression and the fate of marginally viable tissue in the setting of cerebral ischemia. Future studies will allow us to address route of entry into the brain parenchyma. The study also does not aim to establish exposure-response curves at different nPM concentrations or exposure durations. Suture size and ischemic time were chosen to generate small, subcortical strokes in order to optimize the impact of the detrimental exposure. Particulate matter may impact permanent ischemia differently. In the absence of reperfusion, the dynamics of microvascular failure and its impact on penumbral recruitment changes.

The influence of environmental factors on acute stroke progression is recognized, yet understudied. Our data suggests that prior nPM exposure may increase susceptibility to the detrimental effects of acute ischemic stroke. Further studies should attempt to define minimum deleterious exposure durations and concentrations, and further assess the mechanisms by which nPM exposure impacts stroke progression. Once these parameters are better defined, translational studies will be needed to characterize particulate matter exposures and clinical outcomes for susceptible patients at high risk for ischemic stroke.
